# The Zamvar pericardial fold

**DOI:** 10.1186/s13019-017-0641-1

**Published:** 2017-09-12

**Authors:** Vipin Zamvar

**Affiliations:** Royal Infirmary of Edinburgh, Edinburgh, EH14 1JJ UK

## Abstract

This paper describes a pericardial fold that has not yet been mentioned in the Anatomy or Surgery literature.

The “Zamvar” pericardial fold is formed by the parietal pericardium and the overlying fibrous pericardium folding back onto themselves over the left-sided pulmonary veins; it is 1 to 3 mm wide, and runs from the inferior edge of the left inferior pulmonary vein, to the superior edge of the left superior pulmonary vein. A similar fold is not seen on the right side.

The presence of this fold allows for the safe placement of the deep pericardial retraction suture used during off-pump coronary artery surgery.

This paper describes a pericardial fold, which has not yet been mentioned in the Anatomy or Surgery literature. The surgical significance of the pericardial fold is described.

**ANATOMY of the Pericardium**: The pericardium is the sac containing the heart and the roots of the great vessels. The pericardium is made up of two layers: the outer layer known as the “fibrous” pericardium, and the inner layer known as the “serous” pericardium [1].

The fibrous pericardium is the tough external layer; while the serous pericardium is the thin internal layer.

FIBROUS PERICARDIUM:

The fibrous pericardium is made up of tough connective tissue and this makes it relatively non-distensible.

The fibrous pericardium is conical in shape, and it fuses with and is continuous with the following: [1]

1. Inferiorly: with the central tendon of the diaphragm
2. Supero-posteriorly: with the adventitial layers of the aorta, pulmonary artery, and the superior vena cava,
3. On the sides with the adventitial layer of the pulmonary veins,
4. Anteriorly: with the sternopericardial ligaments
5. Superiorly with the pre-tracheal fascia

SEROUS PERICARDIUM:

The serous pericardium is enclosed within the fibrous pericardium. The serous pericardium is divided into two layers: the outer parietal layer, and the inner visceral layer.

The outer parietal layers lines the internal surface of the fibrous pericardium and the internal visceral layer forms the outer layer of the heart (also known as the epicardium).

Each of the layers of the serous pericardium is made up of a single sheet of epithelial cells, known as “mesothelium”.

During development, the heart invaginates the serous pericardium, and it is thus that the two layers of the serous pericardium are formed.

The parietal and visceral layers of the pericardium are in continuity at the points at which the great vessels traverse the pericardial cavity.

PERICARDIAL CAVITY:

Between the visceral and parietal layers of the serous pericardium is the pericardial cavity. It contains a small amount of lubricating serous fluid. This fluid minimises the friction generated by the heart as it contracts and moves about within the pericardial cavity.

PERICARDIAL SINUSES: (Fig. 1).

**Fig. 1:**
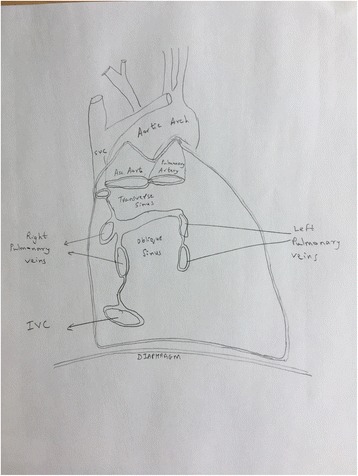
Pericardial Sinuses. The heart has been removed. This figure shows the location of the Transverse pericardial sinus, and the oblique pericardial sinus

As described earlier, the visceral layer of the serous pericardium (also called epicardium) surrounds the heart and great vessels, and the parietal layer lines the fibrous component. The reflections of the serosal layers of the pericardium are arranged around two tubes. One tube encloses the aorta and the pulmonary artery. The second tube encloses the superior vena cava, the inferior vena cava, and the four pulmonary veins.

The **Transverse Pericardial Sinus** is the passage between these two tubes.

It is located

- Posteriorly to the aorta and the pulmonary trunk,
- Anteriorly to the superior vena cava, and
- Superiorly to the left atrium
  The Transverse Pericardial Sinus is also called the **Theile canal** after the German anatomist, Friedrich Theile (1801–1879).

The **Oblique Pericardial Sinus** is a recess in the pericardial cavity situated posteriorly to the heart, and is bounded laterally by the pericardial reflections on the pulmonary veins and the inferior vena cava and posteriorly by the pericardium overlying the anterior aspect of the oesophagus.

**The vestigial fold of the Pericardium**: Between the left pulmonary artery and the left superior pulmonary vein, is a triangular fold of the serous pericardium, also called as the vestigial fold of Marshall (named after John Marshall, British anatomist and surgeon, 1818 to 1891). It is formed by the serous pericardium folding back upon itself over the remnant of the lower part of the left superior vena cava. (also known as the duct of Cuvier, named after Georges Cuvier, French Naturalist, and Zoologist, 1769 to 1832).

## The ZAMVAR pericardial fold (Fig. 2)

As mentioned earlier, the serous pericardium is made up of two layers, the visceral pericardium which lines the surface of the heart, and the parietal pericardium which lines the under-surface of the fibrous pericardium.

**Fig. 2:**
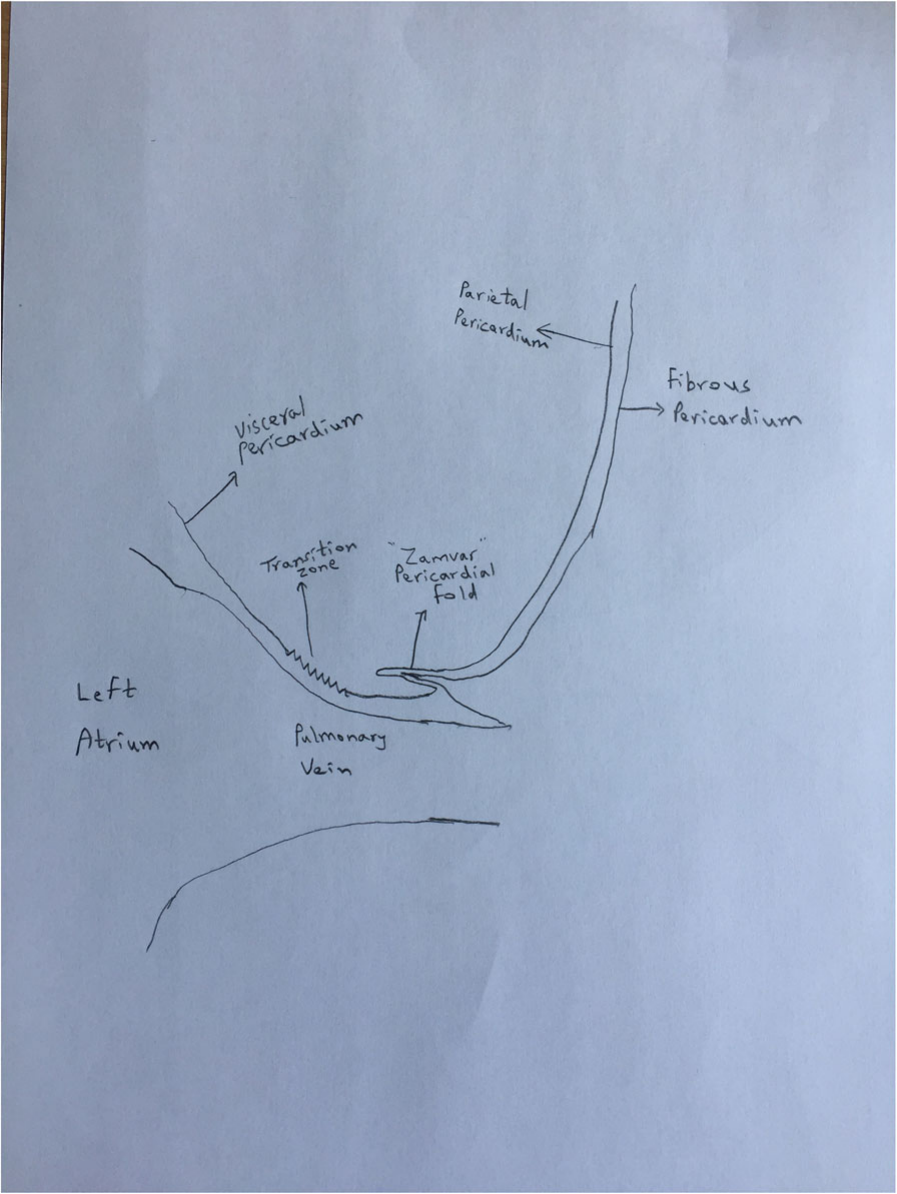
The Zamvar Pericardial Fold. This figure shows a cross-sectional view of the anterior surface of the left sided pulmonary vein. The visceral pericardium covering the left atrium, becomes the parietal pericardium lining the under-surface of the fibrous pericardium. It folds back onto itself forming the “Zamvar” Pericardial Fold

These two layers are continuous with each other over the major arteries and veins as they enter/exit the heart.

The visceral pericardium covers the heart, and where the major vessels enter/exit the heart, it becomes the parietal pericardium.

A special situation arises over the left-sided pulmonary veins.

**Fig. 3:**
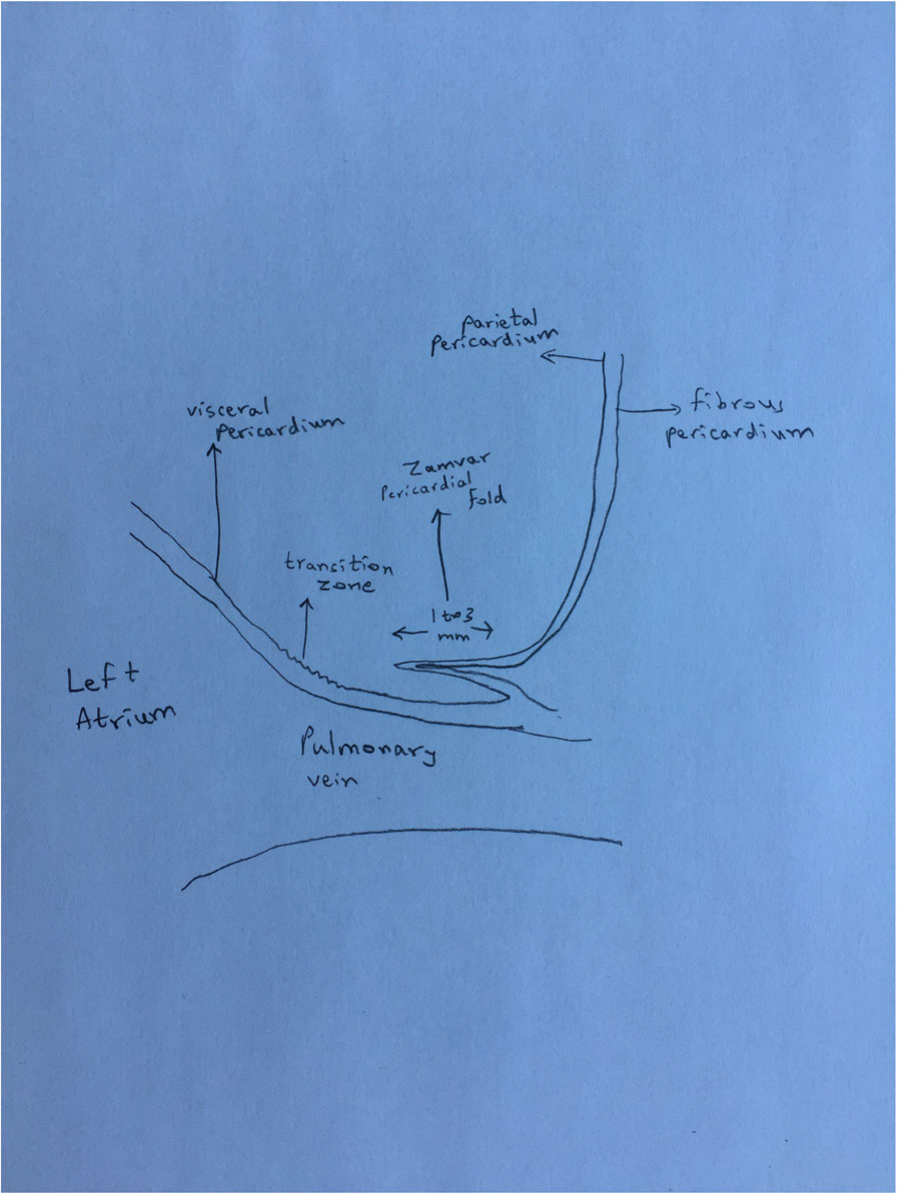
Width of the “Zamvar” Pericardial Fold. The width of the “Zamvar” Pericardial Fold ranges from 1 to 3 mm. It is constituted of the visceral pericardium as well as the underlying fibrous pericardium, which provides it with relative toughness

As shown in in Fig. 2, the transition zone is the region where the visceral pericardium becomes the parietal pericardium. After becoming the parietal pericardium, the serous pericardium lies on the under-surface of the fibrous pericardium. The serous pericardium is quite fused with the fibrous pericardium. The serous pericardium and the fibrous pericardium fold onto themselves over a distance of 1 to 3 mm (Fig. 3), and this forms the newly described “Zamvar” pericardial fold. This fold runs longitudinally from the inferior edge of the inferior pulmonary vein, to the superior edge of the superior pulmonary vein.

**Fig. 4:**
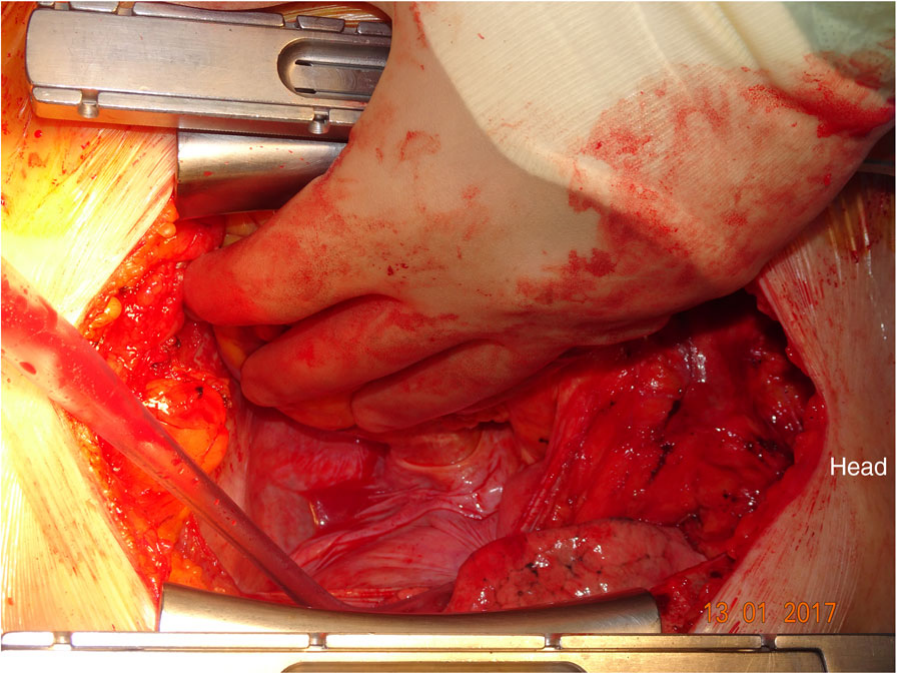
Intraoperative Photograph showing the “Zamvar” Pericardial Fold. Median sternotomy has been performed. The pericardium is opened. The surgeon standing on the patient’s right has lifted the heart using his left hand. The beating heart is lifted off the posterior pericardium, and is pushed into the right pleural cavity. The top of the photo corresponds to the right side of the patient, and the patients head is towards the right side of the photo

**Fig. 5:**
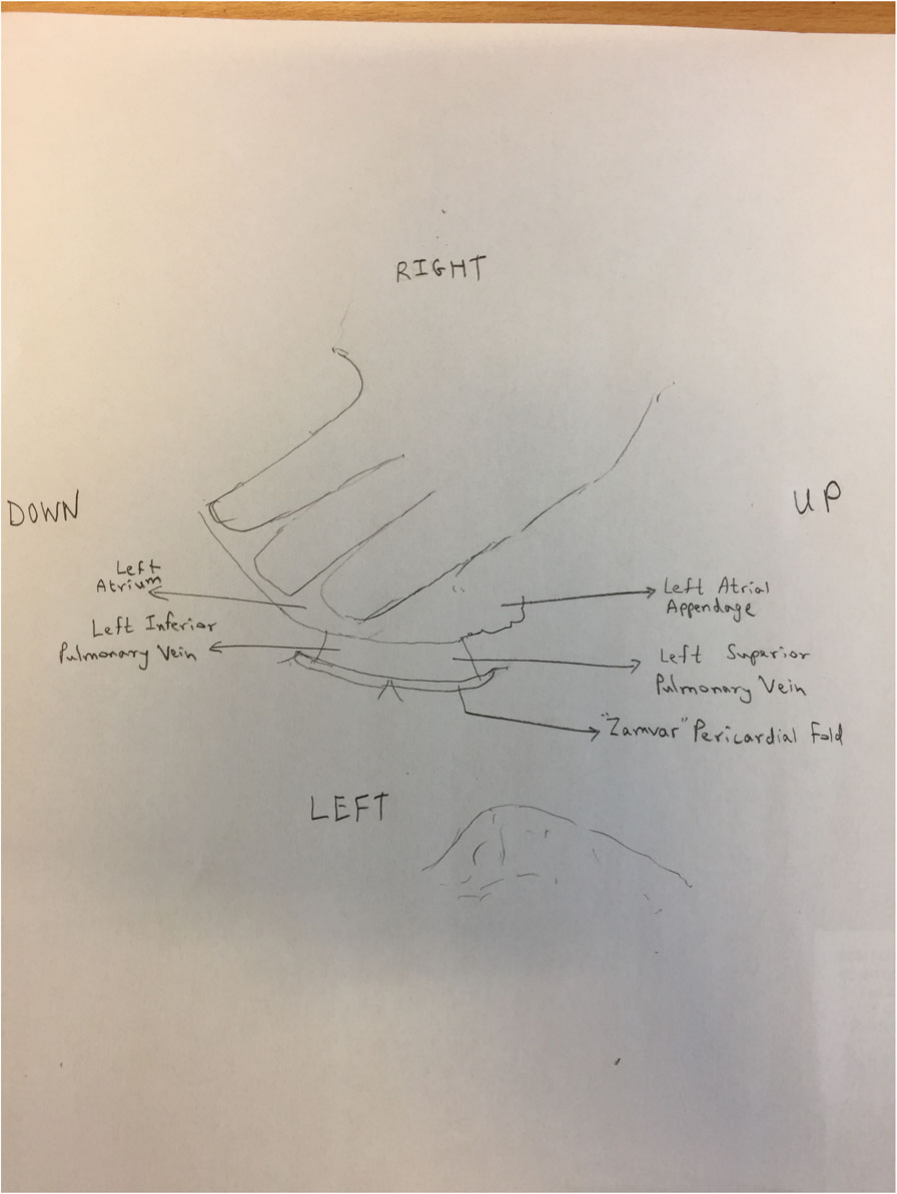
Line Diagram corresponding to Fig. 4. The surgeon’s left hand has lifted the beating heart off the posterior pericardium, and the left atrium is under the surgeon’s fingers. The left atrial appendage can be seen in the right corner. The two left sided pulmonary veins can be seen exiting the left atrium. The pericardial fold is shown in this figure, and it runs from the inferior edge of the left inferior pulmonary vein to the superior edge of the left superior pulmonary vein

Figure 4 is an intra-operative photograph, and Fig. 5 is the corresponding line-diagram explaining the various landmarks. Figures 4 and 5 show the pericardial fold. The photograph has been taken during the conduct of off-pump coronary artery bypass graft surgery. The pericardium has been opened, and the beating heart has been lifted off the posterior pericardium, and has been displaced to the right.

The surgeon is standing on the right side of the patient, and with his left hand has lifted the heart off the posterior pericardium. The apex in moved up and to the right. The surgeon’s left hand covers the posterior surface of the heart, and the left atrium, The left atrial appendage is seen in the corner. The two pulmonary veins are seen exiting the left atrium. The “Zamvar” pericardial fold is clearly seen running from the inferior edge of the left inferior pulmonary vein to the superior edge of the left superior pulmonary vein.

**Fig. 6:**
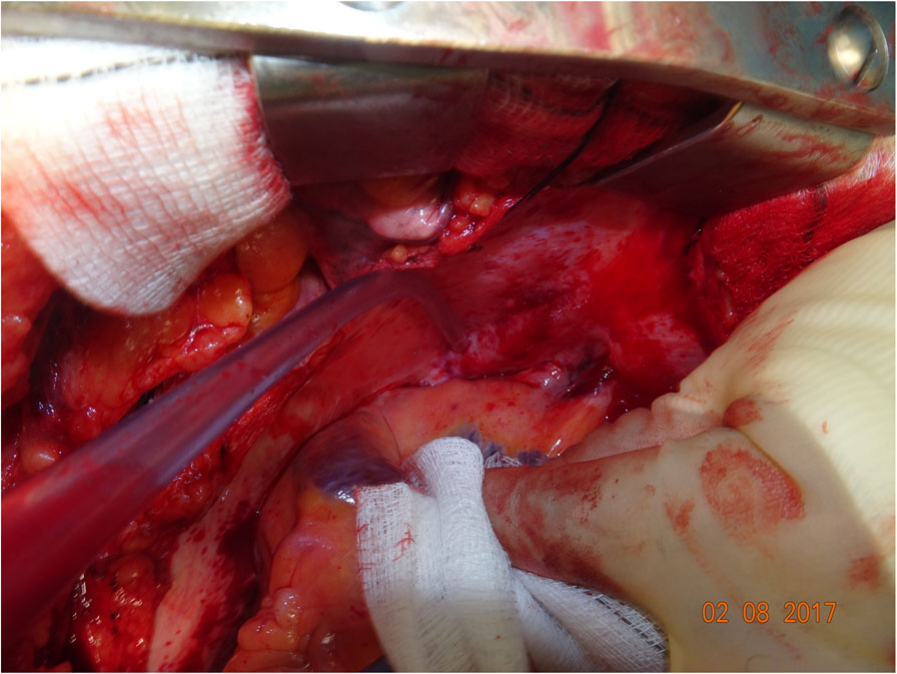
Intraoperative Photograph showing right sided pulmonary veins. This is an intraoperative photograph showing the right sided pulmonary veins. There is no similar fold on the right side

**Fig. 7:**
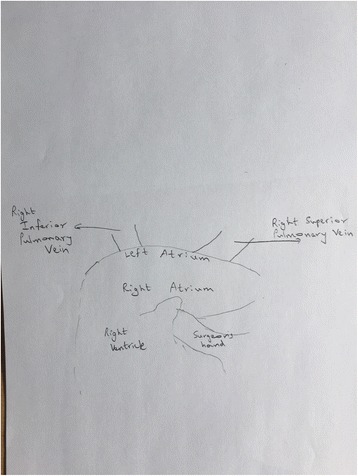
Line diagram corresponding to Fig. 6. It shows the right superior pulmonary vein. There is no pericardial fold over the right-sided veins

Figure 6 is an intra-operative photograph, and Fig. 7 is the corresponding line diagram, showing the various landmarks. These figures show the pulmonary veins on the right side. There is no similar fold on the right side.

**Fig. 8:**
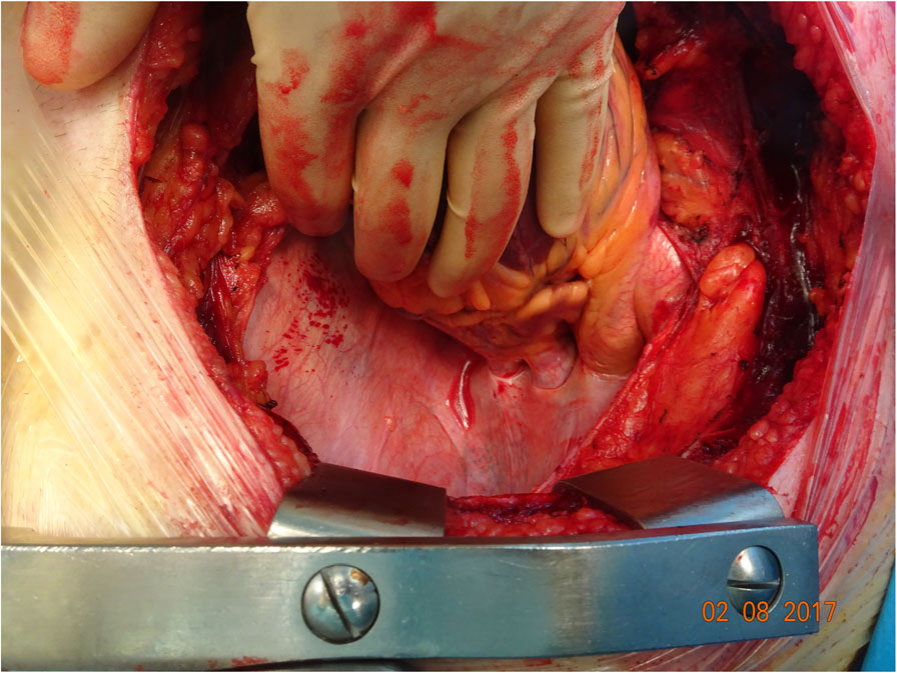
The “Zamvar” Pericardial Fold seen even when the left pleura is intact. This photograph shows the beating heart being retracted to the right, and the “Zamvar” pericardial fold is clearly seen. In this patient the left pleura is not open. The presence of this fold is not dependent on the pleura being open

The presence of this fold on the left side is not dependent on whether the pleura is open or not. Figure 8 is an intraoperative photograph showing clearly the “Zamvar” pericardial fold; in this case the left pleura is intact.

## Surgical significance of the “Zamvar” pericardial fold:

During conduct of off-pump coronary artery surgery, a deep pericardial retraction suture is used to lift the pericardium (and hence the heart) up. Along with other manoeuvres, pulling the pericardium up with a deep pericardial retraction stitch is an essential step to achieve hemodynamic stability and aid access to the deeper parts of the coronary circulation.

**Fig. 9:**
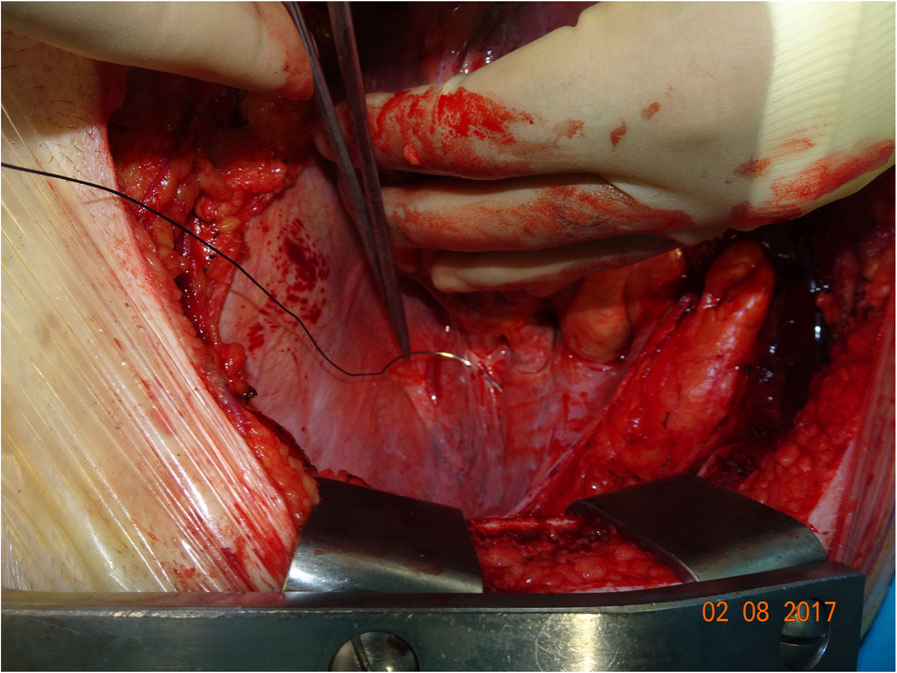
Pericardial Stitch being taken through the “Zamvar” Pericardial Fold. This photograph shows the deep pericardial retraction suture being taken across the “Zamvar” pericardial fold

A number of ways of taking the deep pericardial stitch have been described [2–6]. These include the Lima stitch [2] and its various modifications [3–6]. While experimenting with various positions for the optimal positioning of the pericardial stitch, the author serendipitously came across the anatomical feature of the “Zamvar” pericardial fold, and found that a stitch placed across the pericardial fold across the body of the left inferior pulmonary vein was best able to provide lifting of the pericardium, which in addition to other surgical manoeuvres provided optimal hemodynamic stability during beating heart surgery (Fig. 9).

Over the last 15 years, in more than a thousand coronary artery bypass graft operations, the author has used a deep pericardial retraction stitch placed on the “Zamvar” pericardial fold. He calls it the “Zamvar” pericardial stitch.

This stitch placed across the pericardial fold running over the inferior pulmonary vein allows for the heart to be stabilised with tissue stabilisers (The Octopus stabiliser is the stabiliser of choice for the author; but many other proprietary stabilisers are also available).

The author has found that placing a stitch across the body of the inferior pulmonary vein is the most optimal position for placing this stitch. Also, only one stitch is required, without the need for extra stitches, or gauze pieces [2–6].

The presence of fibrous pericardium in the pericardial fold affords strength to the pericardial fold, and therefore allows the stitch to be taken without the risk of cutting through.

The presence of this fold through which the stitch is taken also protects from inadvertent passing of the needle through the inferior pulmonary vein.

This stitch is also useful in retracting the pericardium upwards to expose the obtuse marginal arteries, during on-pump coronary artery surgery.

## Conclusion

This paper describes a pericardial fold which has not yet been described in the Anatomy or Surgery literature. The presence of this fold allows the placement of a stitch across the anterior surface of the body of the left inferior pulmonary vein, which is an important adjunct to the safe conduct of beating-heart coronary artery bypass graft surgery. It can also be used to lift the pericardium up to facilitate exposure of the obtuse marginal arteries during on-pump coronary artery surgery.
